# Lipid composition of circulating multiple-modified low density lipoprotein

**DOI:** 10.1186/s12944-016-0308-2

**Published:** 2016-08-24

**Authors:** E. R. Zakiev, V. N. Sukhorukov, A. A. Melnichenko, I. A. Sobenin, E. A. Ivanova, A. N. Orekhov

**Affiliations:** 1 Laboratory of Angiopathology, Institute of General Pathology and Pathophysiology, 125315 Moscow, Russia; 2 Department of Development and Regeneration, KU Leuven, 3000 Leuven, Belgium; 3 Institute for Atherosclerosis Research, Skolkovo Innovative Center, 121609 Moscow, Russia

**Keywords:** Low density lipoprotein, LDL, LDL modification, Lipid composition of LDL, Atherosclerosis

## Abstract

Atherogenic modified low- density lipoprotein (LDL) induces pronounced accumulation of cholesterol and lipids in the arterial wall, while native LDL seems to lack such capability. Therefore, modified LDL appears to be a major causative agent in the pathogenesis of atherosclerosis. Possible modifications of LDL particles include changes in size and density, desialylation, oxidation and acquisition of negative charge. Total LDL isolated from pooled plasma of patients with coronary atherosclerosis, as well as from healthy subjects contains two distinct subfractions: normally sialylated LDL and desialylated LDL, which can be isolated by binding to a lectin affinity column. We called the desialylated LDL subfraction circulating modified LDL (cmLDL). In this study, we focused on lipid composition of LDL particles, analysing the total LDL preparation and two LDL subfractions: cmLDL and native LDL. The composition of LDL was studied using thin-layer chromatography. We found that cmLDL subfraction had decreased levels of free and esterified cholesterol, triglycerides, phospholipids (except for lysophosphatidylcholine) and sphingomyelin in comparison to native LDL. On the other hand, levels of mono-, and diglycerides, lysophosphatidylcholine and free fatty acids were higher in cmLDL than in native LDL. Our study demonstrated that lipid composition of cmLDL from atherosclerotic patients was altered in comparison to healthy subjects. In particular, phospholipid content was decreased, and free fatty acids levels were increased in cmLDL. This strengthens the hypothesis of multiple modification of LDL particles in the bloodstream and underscores the clinical importance of desialylated LDL as a possible marker of atherosclerosis progression.

## Background

Atherosclerosis is triggered by lipid accumulation in subendothelial arterial cells [1]. Intracellular lipid accumulation is caused by low-density lipoprotein (LDL) circulating in human blood. It has been demonstrated that only modified lipoprotein, but not native LDL, can cause intracellular lipid accumulation in human arterial wall cells [2]. Although oxidation remains the most studied atherogenic modification of LDL, other modified LDL forms were detected in the blood of atherosclerotic patients. These modifications have not received much attention so far, but may be highly relevant for the development of atherosclerosis. Study of the total LDL from the blood of atherosclerotic patients revealed the presence of desialylated LDL particles with atherogenic properties [2]. Glycosphingolipids in LDL have a terminal sialic acid residue. After removal of sialic acid, galactose becomes the terminal saccharide. We took advantage of this fact to isolate the subfraction of desialylated LDL from total LDL preparation using *Ricinus communis* agglutinin (RCA_120_), which possesses a high affinity to the terminal galactose. Total LDL preparation was applied on a column with RCA_120_ immobilized on CNBr-activated agarose. Normally sialylated LDL passed through the column without binding to the sorbent. Desialylated LDL was bound to lectin and then eluted with 5–50 mM galactose. This method allowed us isolating subfactions of both sialylated and desialylated LDL from the total LDL preparation obtained from the blood of atherosclerotic patients. The latter subfraction is referred to as circulating modified LDL (cmLDL) [2]. In this work we focused on lipid composition of cmLDL particles in comparison with native LDL.

## Methods

### Study subjects

Study subjects included men and women aged 30 to 60 with angiographically demonstrated atherosclerosis and healthy donors aged 25 to 55 with no signs of ischemic heart disease according to Rose questionnaire [3, 4]. The study was approved by the local ethics committee of the Institute of General Pathology and Pathophysiology (Moscow, Russian Federation). Cholesterol and triglycerides in blood plasma of study subjects did not exceed 200 mg/dl and 150 mg/dl respectively. Individuals with diabetes and arterial hypertension were excluded from the study.

### Preparation of blood plasma and serum

Blood samples were drawn in the morning after a night of fasting using tubes with 1 mg/ml EDTA, 0.38 % sodium citrate or 10 u/ml sodium heparin (final concentrations). Samples were centrifuged for 10 min at 800 g in 5 mM of phenylmethylsulfonyl fluoride (PMSF), 1 mM ε-aminocapronic acid and 0.01 % sodium azide. Serum was obtained by incubation of blood samples for 1 h at 37 °C followed by centrifugation for 15 min at 800 g.

### Total LDL preparation

Total LDL fraction was prepared as described previously [5] or using the method established by our group. 8 ml of plasma or serum was overlayed by 2 ml of NaBr solution with density 1.019 g/ml in isotonic phosphate buffer in a centrifuge tube and centrifuged first for 18 h at 12 °C (40000 rpm using 50Ti rotor, Beckman Instruments, USA). 2 ml of the upper fraction was accurately collected and the remaining volume was brought to the density of 1.070 g/ml with solid NaBr. Next, 2 ml of NaBr solution with density 1.065 g/ml was accurately layered on top of the resulting solution and the tube was centrifuged for 24 h at 12 °C (40000 rpm). 2.5 ml of solution containing LDL were collected from the top of the tube and dialysed against 6000 volumes of phosphate buffer with 10 μM EDTA for 24 h. The solution was filter-sterilized and stored in plastic tubes at 4 °C for 1 to 2 weeks. We have developed a faster (4.5 – 5 h) method of total LDL extraction. Blood plasma was brought to density of 1.390 g/ml with solid NaBr (2 g/ml of plasma), and 4 ml of plasma was put into a centrifuge tube. 6 ml of NaBr solution with density 1.019 g/ml was layered on top and the tube was centrifuged for 2 h (40000 rpm). The upper 2 cm of liquid containing floating LDL were collected, 2 g/ml of NaBr was added to the solution, and the tube was centrifuged again in identical conditions. The obtained LDL samples were dialyzed and stored as described above. The LDL fractions obtained by the two methods had similar physical and chemical properties [6].

### Lectin chromatography of modified LDL

ApoB contains 2 types of oligosaccharide conjugates: oligomannoside and terminally sialylated [7, 8]. Glycosphingolipids in LDL also have a terminal sialic acid residue [9]. Desialylation will result in the exposure of the next residue of the carbohydrate chain, which is galactose. Correspondingly, we hypothesized that desialylated LDL will interact with galactose-specific lectins, such as *Ricinus communis* agglutinin (RCA_120_) [10]. To prepare the affinity columns, RCA_120_ was immobilized on CNBr-activated agarose as described earlier [11]. The columns were equilibrated with 10–15 ml of isotonic phosphate buffer, pH 7.2, and 0.5 – 5 ml of LDL sample (containing 0.2 to 10 mg of protein) was loaded on the column. The major part of unbound LDL was washed from the column in the first 10 volumes of phosphate buffer. Desialylated LDL was washed from the column with galactose solutions in phosphate buffer (5, 10, 20, 50 and 100 mM). Washing with 50 mM galactose resulted in virtually complete elution of LDL in the first 5 volumes and this concentration was used most often [12]. Bound and unbound LDL fractions were brought to the density of 1.070 g/ml with NaBr, concentrated by ultracentrifugation and dialyzed against 6000 volumes of isotonic phosphate buffer as described above.

### Lipid analysis

Total lipids were extracted from LDL using a previously described method [13] in chloroform-methanol 1:2 (v/v) mixture. 20 μl of ^14^C-labelled cholesterol in methanol (1 μCi/ml) was added to samples for future low correction. 450 μl of chloroform:ethanol solution in 1:2 ratio (volumetric) were added to samples and vigorously shaked afterwards. Then the samples were incubated in tubes with closed lids for 2 h at room temperature. The mixtures were then centrifuged at 4500 rpm for 15 min and supernatant was transferred to another tube. Residue was resuspensed in 120 μl of distilled water. 450 μl of chloroform:methanol in 1:2 ratio solution was added and lipid extraction and centrifugation routine was repeated as described above. Supernatants were combined and 600 μl of a mix of chloroform:water 1:1 ratio (volumetric) was added. The samples were then vigorously shaken and centrifuged for 10 min at 4500 rpm. Then chloroform phase was extracted, diluted with benzol and evaporated in vacuum in exicator till dry residue left, which was subsequently dissolved in 50 μl of mix chloroform:methanol in 2:1 ratio (volumetric) and stored in closed glass tubes at −20 °C till the analysis of lipid content of the samples was conducted. Main classes of lipids in LDL were determined using thin-layer chromatography. Lipids extracted from LDL and lipid standards. In order to quantify losses we applied 20 μl of a ^14^C labelled cholesterol in methanol solution (1 μCu/ml). Chromatography for neutral lipids was conducted in a hexane – diethyl ester – acetic acid 80:20:1 (volumetric) workflow in a glass chamber filled with solvent vapour for vertical thin-layer chromatography with ground-in lid. Then the plate was scanned on Shimadzu CS-930 (Shimadzu, Japan) densitometer at 200 nm wavelength and lipid content was calculated based on the area of peaks. Next, neutral lipids were eluted twice up to the top of the plate using hexane-acetone in 1:3 volumetric ratio and then again scanning at 200 nm wavelength was used. Having determined position of the common peak of the neutral lipids, areas of silicagel concordant with the peak of each sample were scratched out from the plate and transferred into glass scintillation flasks, containing 15 ml of scintillation liquid and put into liquid scintillation counter 1215 Rackbeta LKB (LKB, Sweden). Main lipid classes content of LDL were calculated considering in mind the losses that took place during extraction.

Determination of main phospholipids fractions content in LDL has also been performed using a densitometer. Residual after elimination of neutral lipids phospholipids were eluted in workflow chloroform – methanol – acetic acid – water in 25:15:4:2 ratio (volumetric) and analyzed on Shimadzu CS-930 (Shimadzu, Japan) densitometer at 200 nm wavelength.

## Results

**Fig. 1:**
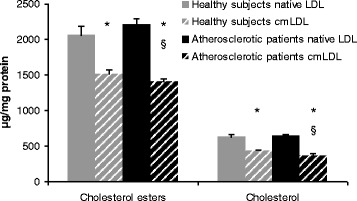
Analysis of cholesterol and cholesterol esters contents in native LDL and cmLDL of healthy donors and coronary atherosclerosis patients. Presented are the means of 3 independent measurements ± standard deviation. *, significant difference from native LDL, *p* < 0.05, §, significant difference from healthy donors, *P* < 0.05 (two-tailed Student’s *t*-test)

**Fig. 2:**
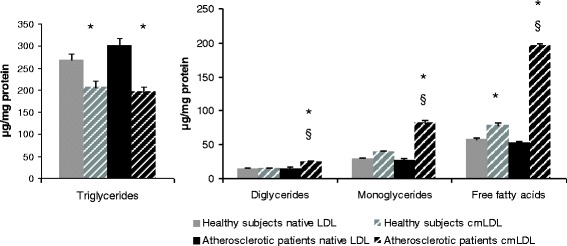
Analysis of main classes of lipids in native LDL and cmLDL of healthy donors and coronary atherosclerosis patients. Presented are the means of 3 independent measurements ± standard deviation. *, significant difference from native LDL, *P* < 0.05, §, significant difference from healthy donors, *p* < 0.05 (two-tailed Student’s *t*-test)

**Fig. 3:**
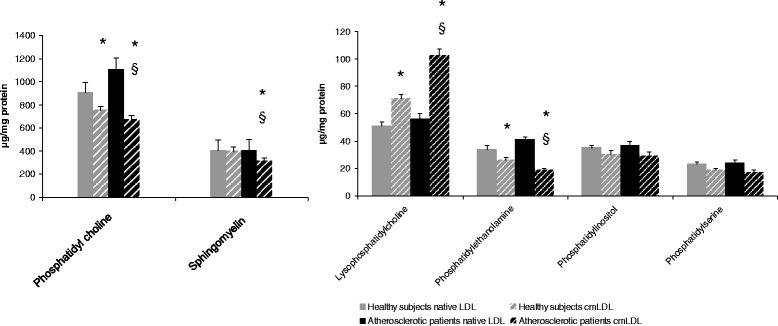
Analysis of main classes of phospholipids in native LDL and cmLDL of healthy donors and coronary atherosclerosis patients. Presented are the means of 3 independent measurements ± standard deviation. *, significant difference from native LDL, *P* < 0.05, §, significant difference from healthy donors, *p* < 0.05 (two-tailed Student’s *t*-test)

We performed quantitative analysis of the main classes of neutral lipids in native LDL and cmLDL from healthy donors and coronary atherosclerosis patients. In cmLDL from healthy donors, free and esterified cholesterol and triglycerides levels were 30 – 40 % lower in comparison to native LDL (Fig. 1). On the other hand, monoglycerides and free fatty acid levels in cmLDL were 1.5-fold higher than in native LDL (Fig. 2). In cmLDL from atherosclerotic patients, contents of free and esterified cholesterol and TGs were 1.5 to 2 fold lower, and levels of mono- and diglycerides and free fatty acids – 3 to 4 fold higher than in native LDL. cmLDL from atherosclerotic patients contained less phosphatidylcholine, phosphatidylethanolamine and sphingomyelin and more lysophosphatidylcholine than native LDL (Fig. 3). Levels of major classes of phospholipids in native LDL from atherosclerotic patients were similar to those in native LDL from healthy donors.

## Discussion

Desialylation is likely to be the first or one of the first steps in the chain of multiple atherogenic modifications of LDL particles in the bloodstream of atherosclerotic patients [2]. Incubation of native LDL with blood plasma obtained from atherosclerotic patients results in a decrease of the sialic acid content of lipoprotein particles as soon as after 1 h (Table 1). Desialylation is followed by other physical and chemical modifications including decrease of the cholesterol, phospholipids and triglycerides contents, decrease of the particle size and increase of the electrical charge (Table 1). Finally, LDL particles are oxidized (the level of apoB-bound cholesterol is increased), which can be explained by possible increased susceptibility of modified LDL particles to oxidation resulting from a decreased content of natural antioxidants such as vitamin E (Table 1). LDL atherogenicity, hence the ability to induce lipid accumulation in cultured smooth muscular cells and macrophages, increased with time during the incubation [14]. LDL acquired atherogenic properties already after 3 h of incubation with blood plasma from atherosclerotic patients (Table 1), and its atherogenic potential increased during the incubation until 36 h, when the maximum was reached [14]. Based on these observations, it can be proposed that multiple modification of LDL is a cascade of changes of the lipoprotein particle, which is initiated by desialylation followed by the decrease of lipid contents and change of physical parameters (decrease of particle size, increase of density and electronegativity) and finalized by oxidation. Every consequent change of the LDL particle increases its atherogenicity. It is, however, likely that the input of oxidation to the increase of the LDL atherogenic potential is minimal.

**Table 1:** Scheme of LDL modification

1 h	3 h	6 h	12 h	24 h	36 h	48 h
↓	↑	↓	↓	↓	↑	↑
Sialic acid		Free cholesterol	Size	Triglycerides	Electronegativity	Apo B-bound cholesterol
↑			↓			↑
% of desialylated LDL			Cholesteryl esters			Susceptibility to oxidation
			↓			↑
			Phospholipids			Fluorescence
						↓
						Vitamin E

Adapted from [14] with permission

**Fig. 4:**
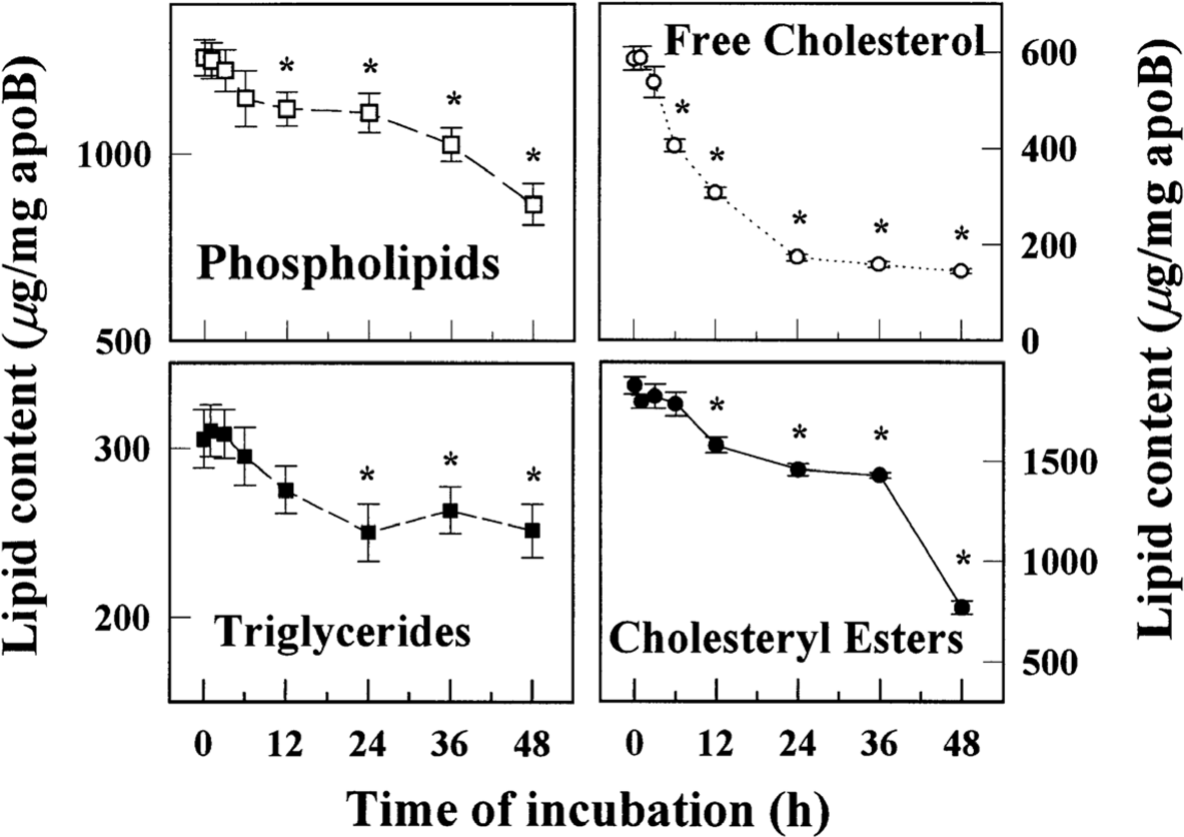
Contents of phospholipids, triglycerides, free and esterified cholesterol in LDL incubated with plasma-derived serum at 37 °C. Data are the means of three determinations ± S.E.M. *Significant difference from non-incubated LDL, *P* < 0.05. Adapted from [14] with permission

In this work, we analyzed the lipid composition of native and desialylated (multiple-modified) LDL isolated from the blood of atherosclerotic patients. We compared specifically normally sialylated (native) and desialylated LDL. Native and desialylated LDL particles are present in the blood of both healthy individuals and atherosclerotic patients. It is important to study the differences of lipid composition of native and deslaylated LDL in healthy individuals and atherosclerotic patients. It was demonstrated that the contents of free and esterified cholesterol, triglycerides and most of the phospholipids was decreased in multiply modified LDL in comparison to native LDL. At the same time, the contents of mono- and diglycerides and free fatty acids was increased, probably, as a result of disintegration of triglycerides and other complex lipids. Importantly, changes of the lipid composition of naturally occurring circulating multiple-modified LDL are identical to changes occurring during incubation of native LDL with blood plasma of atherosclerotic patients. Such incubation also results in a decrease of the contents of free and esterified cholesterol, triglycerides and phospholipids (Fig. 4). This strengthens the hypothesis that multiple modification of LDL takes place in the bloodstream.

It has been demonstrated that lipid accumulation in the arterial wall cells triggers atherogenesis at the cellular level [2]. Intracellular lipid accumulation is accompanied by the pro-inflammatory immune response, enhanced proliferation and extracellular matrix synthesis leading to atherosclerotic lesion development and progression [2]. Taking into account that only multiply-modified, but not native LDL, causes intracellular lipid accumulation, it can be concluded that atherosclerosis development is dependent not so much on the total LDL level, as on the content of modified LDL. Analytical methods aimed to measure the level of different forms of modified LDL in the blood have been developed [15]. However, these methods have not been implemented in clinical practice so far.

Currently, oxidation is widely regarded as the main, if not the only form of LDL modification that leads to formation of atherogenic LDL [16–18]. Our data challenge this point of view. First, atherogenic properties of LDL can be associated with other types of modification. Second, each consequent modification of LDL increases its atherogenic potential. Third, oxidation is one of the latest atherogenic modifications in the chain. Fourth, oxidation does not increase atherogenic potential of multiple-modified LDL particles any further.

Therefore, multiple modification of LDL is likely to be the cause of its atherogenicity. Modification of the lipid composition of LDL represents only one of several aspects of atherogenic multiple modification of LDL.

## Conclusion

The present study demonstrated that cmLDL isolated from the blood of atherosclerotic patients differs significantly from native LDL by its lipid composition. In particular, cmLDL was characterized by a relative decrease of free and esterified cholesterol, triglyceride and phospholipid content and increase of mono- and diglyceride as well as free fatty acid content.
